# Feasibility and safety of upper limb extremity ergometer exercise in the cardiac intensive care unit in critically ill patients with cardiac disease: a prospective observational study

**DOI:** 10.3389/fphys.2025.1448647

**Published:** 2025-03-28

**Authors:** Kotaro Hirakawa, Atsuko Nakayama, Takeshi Arimitsu, Kazuki Kon, Hiromichi Ueki, Kentaro Hori, Yuki Ishimoto, Ayaka Ogawa, Ryosuke Higuchi, Yumiko Hosoya, Mamoru Nanasato, Mitsuaki Isobe

**Affiliations:** ^1^ Department of Rehabilitation, Sakakibara Heart Institute, Tokyo, Japan; ^2^ Department of Cardiology, Sakakibara Heart Institute, Tokyo, Japan; ^3^ Division of Nurse, Sakakibara Heart Institute, Tokyo, Japan; ^4^ Sakakibara Heart Institute, Tokyo, Japan

**Keywords:** upper limb extremity ergometer, cardiogenic shock, cardiac disease, cardiac intensive care unit, cardiac rehabilitation

## Abstract

**Background:**

There is no established method for early bed rehabilitation of patients after cardiogenic shock (CS) who require mechanical circulatory support (MCS). This study aimed to evaluate the safety of early upper limb extremity ergometer exercise in critically ill patients with CS or at risk for CS.

**Methods:**

The study was conducted as a prospective, single-center feasibility and observational study. Patients with CS or at risk for CS in the cardiac intensive care unit were enrolled. Upper limb extremity ergometer exercise was performed with alternating intervals of exercise and rest, in parallel with the rehabilitation program focused on early mobilization. A multidisciplinary team was established to determine the criteria for exercise initiation and cessation. Endpoint measures included exercise-related adverse events (circulatory or respiratory failure requiring new medical intervention, inability to continue device support, bleeding requiring hemostatic treatment at the insertion site, and exercise-related skeletal pain), vital signs, and subjective symptoms.

**Results:**

Forty-seven sessions in 28 patients (71 ± 15 years, 75% male) were included in the analysis. MCS was used in 86% of the patients, and rehabilitation was initiated within 3 ± 1 day. Two patients had temporary dyspnea, and none discontinued exercise. There was a significant increase in heart rate (p < 0.05) and respiratory rate (p < 0.001) during exercise compared with pre-excercise, but not in systolic or diastolic blood pressure and oxygen saturation.

**Conclusion:**

Early upper limb extremity ergometer exercises for critically ill patients with CS or at risk for CS resulted in changes in heart rate and respiratory rate during exercise. However, no exercise-related adverse events occurred. Upper limb extremity ergometer exercise can be a new tool of physical therapy in the acute phase of patients after CS or at risk for CS.

## 1 Introduction

In-hospital mortality remains high in critically ill patients with cardiac disease who experience cardiogenic shock (CS) or multiorgan failure requiring mechanical circulatory support (MCS) or continuous renal replacement therapy (CRRT) (Kolte et al., 2014; Jentzer et al., 2019). These critically ill patients require prolonged management in the intensive care unit (ICU), and even if they survive and are discharged, they are at a high risk of developing post-intensive care syndrome (PICS). This syndrome causes physical, cognitive, and psychological dysfunctions, leading to reduced activities of daily living and quality of life after discharge (Needham et al., 2012; Kawakami et al., 2021). In recent years, early rehabilitation and physical intervention have been initiated within the first few days in the ICU, depending on the patient’s resilience and general condition. It has gained attention as a means of preventing PICS (Renner et al., 2023). On the other hand, early rehabilitation has been shown to carry a risk of adverse events such as cardiac arrest, fatal arrhythmias, significant oxygen desaturation, and unplanned extubation (Paton et al., 2023; Schaller et al., 2024). However, few studies have evaluated the safety and efficacy of early rehabilitation using MCS or CRRT in critically ill patients. The femoral approach is often used when a hemodynamic compromise occurs and urgent MCS is initiated. This can make it difficult to get out of bed and perform lower extremity exercises.

Upper limb extremity ergometer exercises have long been used for patients with orthopedic and peripheral arterial diseases (Mendelsohn et al., 2008; Zwierska et al., 2005) and, more recently, for patients with acute stroke and spinal cord injury (da Rosa Pinheiro et al., 2021; Olney et al., 2023). Upper and lower limb extremity ergometer exercises improve cardiopulmonary function and exercise tolerance (Larsen et al., 2016). However, upper limb extremity ergometer exercises produce greater heart rate and ventilatory variabilities than lower limb extremity exercises (Leicht et al., 2008; Castro et al., 2011). In addition, bradycardia and respiratory distress have been reported as endpoints in cardiopulmonary exercise testing using an upper extremity ergometer (Ilias et al., 2009; Eerden et al., 2018). Previous studies have been conducted in healthy participants and post-discharge patients; therefore, the acute-phase safety in critically ill patients with cardiac disease is unclear. We consider that early rehabilitation with clear criteria for initiation and cessation, shared and implemented by a multidisciplinary team, can be safely delivered without causing adverse events.

This study aimed to evaluate the occurrence of adverse events during or after early upper limb extremity ergometer exercise in critically ill patients with CS or at risk for CS and to validate its safety.

## 2 Materials and methods

### 2.1 Participants

This prospective study was conducted at a single cardiovascular center (Higuchi et al., 2023). This study included patients with or at risk of CS who qualified for the Society for Cardiovascular Angiography and Intervention (SCAI) CS classification A to E (Baran et al., 2019) in the cardiac ICU (CICU) of our hospital from June to December 2023. The exclusion criteria were no admission to the CICU; ability to get out of bed, including standing and walking, within 48 h of illness onset; restricted movement of the upper limb extremity, such as fractures or arthrogryposis; bedridden pre-hospitalization; and no consent to participate in this study. Participants were informed verbally in advance regarding the purpose, content, and handling of the survey results, and their written consent was obtained.

The study was conducted in accordance with the guidelines of the Declaration of Helsinki and the Ethical Guidelines for Research in the Department of Life Sciences and Medical Sciences for People and was approved by the Ethics Committee of Sakakibara Heart Institute (approval ID:23-013).

### 2.2 Ergometer exercise

The indications for upper limb extremity ergometer exercises were determined during daily multidisciplinary conferences with cardiologists, nurses, and physical therapists. The following criteria indicated unstable conditions that prevented the initiation of exercise: exacerbation of heart failure (significant decrease in urine output or worsening edema, weight gain, increased pulmonary opacities on chest x-ray, increased cardiothoracic ratio, etc.), new-onset or uncontrolled organ ischemia as determined by blood test data, such as blood enzymes and markers, and diagnostic imaging, active bleeding (hemoglobin ≤7 g/dL), cerebrovascular events in the previous 24 h, fever >38.5°C, no creatine kinase CK/CK-MB peak, significant ST-segment elevation ≥1 mm within 12 h, arrhythmias that disrupt circulatory dynamics, no recent new inotropic drug initiation or dose increase before the start of exercise, fraction of inspiratory oxygen >0.6, and Richmond Agitation-Sedation Scale (RASS) ≤−3 or ≥+2. Ergometer exercises were initiated if the criteria were not met. Additionally, the following criteria were used to indicate exercise cessation: systolic blood pressure <80 mmHg or >140 mmHg, heart rate <50 beats per min or >120 beats per min, respiratory rate <10 breaths per min or >40 breaths per min, saturation of percutaneous oxygen <90%, significant fatigue (The modified Borg scale score ≥5), excruciating pain or discomfort, and electrocardiographic changes suspicious for new major arrhythmia or myocardial ischemia. The ergometer exercise should be stopped if this criterion is met. Some criteria, such as transient changes in vital signs, were closely monitored. In these cases, the exercise could be temporarily paused and safely resumed upon stabilization of the patient’s condition. These criteria for initiation and discontinuation are consistent with relevant guidelines (Unoki et al., 2023; Kimura et al., 2019), and these criteria were adopted to ensure safety during the exercise.

Upper extremity ergometer exercises were performed using Terasu Ergo (Showa Denki Co., Tokyo, Japan). The exercise protocol is that the exercise load ranged from 3 to 20 W, starting at a low intensity and gradually increasing according to hemodynamics, physical findings, and fatigue. Exercise duration consisted of alternating intervals of exercise and rest in a 1:1 ratio, with a goal of three sets of 3–5 min per set for the first session and a maximum of four sets for the next session, for a total of 15–20 min. The exercise was performed once a day until active walking was possible. In cases with SCAI classification A or B in whom MCS was not initiated, the patients could be early mobilized, which is established as standard rehabilitation in the CICU, and upper limb ergometer exercise was performed in conjunction with this. If a device, such as MCS or CRRT, was applied, upper limb ergometer exercise was performed under the supervision of a cardiologist and a clinical engineering technologist.

### 2.3 Endpoint measures

The primary endpoint was exercise-related adverse events of circulatory or respiratory failure requiring new medical intervention within 24 h of exercise, device removal or misalignment that makes continued support difficult, bleeding requiring hemostatic treatment at the insertion site, and exercise-related skeletal pain in the extremities (TEAM Study Investigators and the ANZICS Clinical Tria et al., 2022).

### 2.4 Additional assessments

The following data were collected from medical records, examinations and measurements in the medical treatment. The SCAI classification was determined on admission to the CICU. The decision to initiate MCS was made by a cardiac team led by a cardiologist. Sequential organ failure assessment (SOFA) score was assessed on admission to the CICU. The SOFA score is calculated by scoring respiration (PaO_2_/FiO_2_), coagulation (platelet count), liver function (total bilirubin), circulation (mean blood pressure, catecholamines), central nervous system (Glasgow coma scale), and renal function (creatinine, urine volume) on a scale of 0–4, respectively, for a total score. The Barthel Index was obtained from the patient or family member regarding the situation pre-admission and assessed by a trained physical therapist at discharge. Systolic/diastolic blood pressure, heart rate, percutaneous oxygen saturation, respiratory rate, Borg scale score, and subjective symptoms (chest symptoms, respiratory symptoms, pain) were measured before, during, and after exercise.

### 2.5 Sample size calculation

A *post hoc* power analysis was conducted using G*Power (version 3.1.9.4, University of Düsseldorf, Germany) to determine the required sample size. The analysis indicated that a sample size of N = 21 was necessary to achieve an effect size of 0.5 with a statistical power of 80% at an alpha level of 0.05.

### 2.6 Statistical analyses

Continuous variables are presented as means ± standard deviations and median (interquartile range), and categorical variables are expressed as percentages. The Shapiro–Wilk test was used to verify normal distribution. Clinical characteristics were categorized into Stage B and below and Stage C and above, which are largely separated by severity of CS and intervention. The percentage change in vital signs at each time point during exercise was calculated as follows: (post-exercise − pre-exercise)/pre-exercise × 100 (%). In addition, differences between changes in vital signs during exercise were examined using repeated-measures analysis of variance and pairwise comparisons with Bonferroni correction. The significance level was set at p ˂ 0.05, and all statistical analyses were performed using IBM SPSS Statistics version 22 (IBM Corp., Armonk, NY, United States).

## 3 Results

**FIGURE 1:**
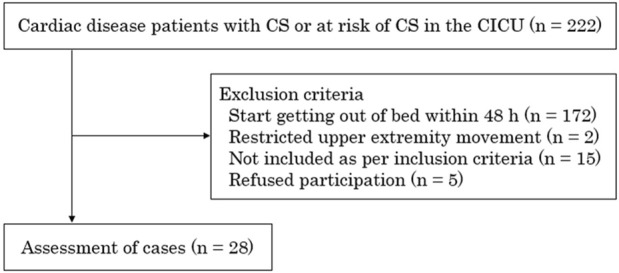
Schematic presentation of the study procedure. This study included 28 patients with or at risk of cardiogenic shock in the cardiac intensive care unit. CS, cardiogenic shock; CICU, cardiac intensive care unit.

Out of 222 patients 28 patients with 47 exercise sessions were included. 194 patients were excluded (172 patients in start to get out of bed in less than 48 h, 2 patients with limited upper extremity movement, 15 patients that did not have the criteria for inclusion, 5 patients refused to participate in exercise) following clinical protocols designed to adapt the exercise prescription according to each patient’s condition and response to therapy (Figure 1). The clinical characteristics of the patients are summarized in Table 1. MCS was used in ≥80% of the cases, including one case of veno-arterial extracorporeal membrane oxygenation (ECMO) combined with Impella.

**TABLE 1:** Clinical characteristics.

Clinical characteristics	Overall (n = 28)	SCAI CS A, B (n = 12)	SCAI CS C, D (n = 16)
Age, years	71 ± 15	78 ± 14	66 ± 14
Male, n (%)	21 (75.0)	8 (66.7)	13 (81.3)
BMI, kg/m^2^	23.2 ± 3.8	22.3 ± 2.6	23.8 ± 4.4
Diagnosis, n (%)			
Acute coronary syndrome	23 (82.1)	10 (83.3)	13 (81.3)
Other	5 (17.9)	2 (17.9)	3 (18.8)
SOFA score	6 ± 3	5 ± 3	6 ± 3
SCAI CS classification, n (%)			
Stage A	8 (28.6)	8 (66.7)	-
Stage B	4 (14.3)	4 (33.3)	-
Stage C	12 (42.9)	-	12 (75.0)
Stage D	4 (14.3)	-	4 (25.0)
Peak CK, U/L	3428 ± 4073	1775 ± 1945	4,668 ± 4,823
Peak CK-MB, U/L	280 ± 292	180 ± 201	360 ± 333
LVEF, %	37.0 ± 13.2	41.3 ± 10.6	33.7 ± 14.3
Post-emergent coronary revascularization, n (%)	22 (78.6)	10 (83.3)	12 (75.0)
V-A ECMO, n (%)	1 (3.6)	0 (0.0)	1 (6.3)
IABP, n (%)	16 (57.1)	8 (66.7)	8 (50.0)
V-A ECMO(%)	12 (42.9)	1 (8.3)	11 (68.8)
IPPV, n (%)	9 (32.1)	5 (41.7)	4 (25.0)
NPPV, n (%)	6 (21.4)	5 (41.7)	1 (6.3)
CRRT, n (%)	5 (17.9)	1 (8.3)	4 (25.0)
Noradrenaline, n (%)	14 (50.0)	3 (25.0)	11 (68.8)
Dobutamine, n (%)	16 (57.1)	4 (33.3)	12 (75.0)
CICU stay, days	15 ± 14	9 ± 3	20 ± 17
Cardiac rehabilitation, days			
Standing	8 ± 8	5 ± 2	11 ± 10
Walking	11 ± 10	7 ± 4	14 ± 12
Bartel Index, points			
Pre-hospital	97 ± 9	94 ± 11	98 ± 6
Discharge	88 ± 26	85 ± 30	90 ± 25
Hospital stay, days	34 ± 26	34 ± 29	34 ± 26
Discharge, n (%)			
Home	23 (82.1)	11 (91.7)	12 (75.0)
Transferred	3 (10.7)	0 (0.0)	3 (18.8)
Death	2 (7.2)	1 (8.3)	1 (6.2)

BMI, body mass index; SOFA, sequential organ failure assessment; SCAI CS, society for cardiovascular angiography and intervention cardiogenic shock; CK, creatine kinase; LVEF, left ventricular ejection fraction; V-A ECMO, veno-arterial extracorporeal membrane oxygenation combined; IABP, intra-aortic balloon pumping; IPPV, invasive positive pressure ventilation; NPPV, non-invasive positive pressure ventilation; CRRT, continuous renal replacement therapy; CICU, cardiac intensive care unit.

**FIGURE 2:**
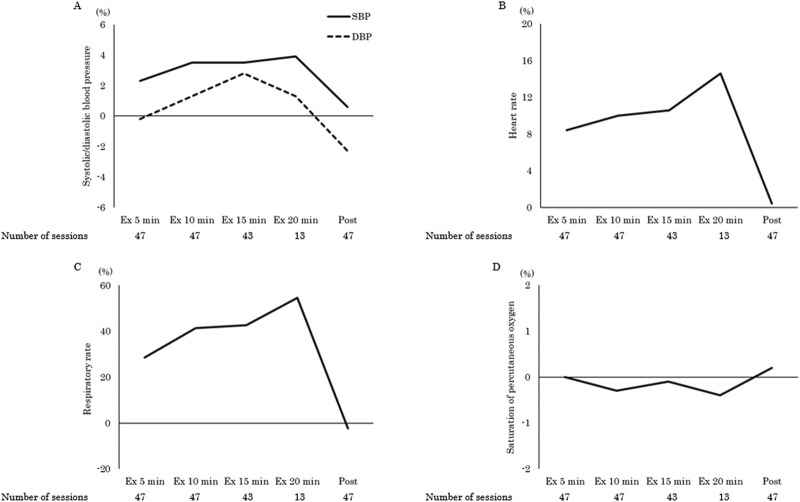
The percentage change in vital signs during and post exercise compared to pre-exercise levels for systolic/diastolic blood pressure **(A)**, heart rate **(B)**, respiratory rate **(C)**, and saturation of percutaneous oxygen **(D)**. SBP, systolic blood pressure; DBP, diastolic blood pressure.

The clincal status during the ergometer exercise period and adverse events are shown in Tables 2, 3. Device management included 68% MCS, 13% mechanical ventilation (MV), and 6% CRRT, with exercise being started at the earliest 3 days after admission to the CICU. In total, 91% of the exercises were performed in a supine or sitting position in bed. The maximum exercise load was 15 (15–20) W, and the exercise duration was 7 (5–10) min. Two patients had temporary dyspnea, one during the exercise and the other after the exercise. None of the patients discontinued the exercise. A one-way repeated measures ANOVA was conducted to examine changes in vital signs across pre-to post-exercise (pre-, 5 min, 10 min, 15 min, 20 min, and post-exercise). Mauchly’s test of sphericity for heart rate indicated that the assumption of sphericity was violated (p < 0.001). Therefore, the Greenhouse-Geisser correction was applied and the adjusted results showed a significant main effect of time (F [1.44, 17.33] = 10.81, p = 0.002, η^2^ = 0.47). Also, adjusted results for the respiratory rate showed a significant main effect of time (F [2.48, 29.80] = 27.59, p < 0.001, η^2^ = 0.70). Post hoc comparisons using Bonferroni correction revealed that both heart rate (82 ± 13 vs. 89 ± 12, 90 ± 13, 91 ± 11, 92 ± 11 beats per minute, p < 0.05) and respiratory rate (21 ± 3 vs. 26 ± 6, 29 ± 7, 29 ± 6, 31 ± 5 breaths per minute, p < 0.001) were significantly higher during exercise than pre-exercise (Table 4). The effect of exercise was greatest for the respiratory rate (percentage change during exercise at 5, 10, 15, and 20 min compared to rest: 28.6, 41.3, 42.6, 54.5%, p < 0.001), followed by the heart rate (8.4, 10.0, 10.6, 14.6%, p < 0.05). Systolic blood pressure (2.3, 3.5, 3.5, 3.9%, p = 0.34), diastolic blood pressure (−0.2, 1.3, 2.8, 1.3%, p = 0.50), and saturation of percutaneous oxygen (0.0, −0.3, −0.1, −0.4%, p = 0.78) remained unchanged (Figure 2).

**TABLE 2:** Clinical status at the time of upper extremity ergometer exercise.

Upper extremity ergometer exercise	47 sessions
First exercise (days), median [IQR]	4 [3–5]
MCS, n (%)	32 (68)
CRRT, n (%)	3 (6)
MV, n (%)	6 (13)
Glasgow Coma Scale, median [IQR]	15 [15–15]
Exercise time (min), median [IQR]	15 [15–20]
Exercise load (W), median [IQR]	7 [5–10]
Positioning, n (%)	
Supine	34 (72)
Sitting (in bed)	9 (19)
Sitting (out of bed)	4 (9)

IQR, interquartile range; MCS, mechanical circulatory support; CRRT, continuous renal replacement therapy; MV, mechanical ventilation.

**TABLE 3:** Incidence of adverse events.

Type of potential adverse events, n (%)	Ergometer exercise	
	During	Post
Hemodynamic change	0 (0)	0 (0)
Temporary dyspnea	1 (2)	1 (2)
Device-related	0 (0)	0 (0)
Bleeding	0 (0)	0 (0)
Pain	0 (0)	0 (0)

**TABLE 4:** Monitoring of vital signs pre-, during, and post-exercise.

Vital signs variables	Pre Ex	Ex 5 min	Ex 10 min	Ex 15 min	Ex 20 min	Post Ex
SBP, mmHg	113 ± 16	116 ± 17	117 ± 17	117 ± 16	116 ± 17	114 ± 17
DBP, mmHg	75 ± 15	74 ± 13	75 ± 15	76 ± 15	71 ± 12	72 ± 14
HR, beats per minute	82 ± 13	89 ± 12^*^	90 ± 13^*^	91 ± 11^*^	92 ± 11^*^	83 ± 13
RR, breaths per minute	21 ± 3	26 ± 6^**^	29 ± 7^**^	29 ± 6^**^	31 ± 5^**^	20 ± 3
SpO_2_, %	98 ± 2	98 ± 2	98 ± 2	98 ± 2	98 ± 1	98 ± 1

*vs. Pre Ex p < 0.05.

**vs. Pre Ex p < 0.001.

SBP, systolic blood pressure; DBP, diastolic blood pressure; HR, heart rate; RR, respiratory rate; SpO_2_, saturation of percutaneous oxygen.

## 4 Discussion

This study is the first report which examines the feasibility and safety of an early initiation of upper limb extremity ergometer exercise in critically ill patients with or at risk for CS. Only 5 out of 222 cardiac disease patients with CS or at risk of CS at the CICU refused participation. No adverse events occurred in the exercising patients requiring additional therapeutic interventions. One out of 28 patients reported dyspnea during or after exercise. There was an increase in heart rate and more pronounced in respiratory rate during exercise compared to pre-exercise values.

Advances in MCS and its treatment have made it possible to establish hemodynamics early after CS, but MCS or MV therapy is necessary for a period of time to restore cardiac function and improve general condition. Therefore, the onset of early mobilization is delayed, and even during this time, muscle catabolism increases, and cardiopulmonary function progressively declines. The few studies on early mobilization in MCS have covered a wide range of subjects, including several diseases, mainly respiratory diseases, and ECMO as a bridge to transplantation patients. Additionally, the timing of exercise initiation was variable, with some patients starting within 2–4 days of disease onset and others taking more than a week. Also direct intervention by multiple healthcare professionals is necessary to ensure safety (Salna et al., 2020; Ferreira et al., 2019; Cucchi et al., 2023). At present, there exists no established guideline or protocol for early bed rehabilitation of post-CS patients who would require MCS support. In this study, standard management with appropriate pain control and light sedation was used, and RASS was included among the criteria for initiating exercise, excluding cases of agitation and delirium. In addition, a total of five patients refused to participate in the exercise, four complaining of discomfort and one who was reluctant to be treated. Furthermore, the median Glasgow Coma Scale score during exercise was 15. This indicates that the patients were able to communicate and could easily express symptoms and complaints. Thus, the introduction of upper limb extremity ergometer exercise in the management of MCS was limited to cases with relatively low anxiety and pain as well as a good arousal status, and it is important to establish such conditioning. In addition, MCS often involves the placement of a catheter in the femoral region, which restricts movement in the lower extremity while the upper extremity is relatively unrestricted. With this in mind, upper extremity ergometer exercises have the advantage of being started early in a supine position, even with MCS support. Furthermore, because it does not require major positional changes, such as out-of-bed changes, the risks of catheter removal and significant dynamic hemodynamic fluctuations are reduced. Additionally, Impella malrotation has been shown to cause severe hemodynamic instability (Baldetti et al., 2023). In this study, exercise was initiated within 3 days, which was considered as an early exercise intervention. In addition, there were no adverse events requiring exercise cessation due to hemodynamic compromise, device removal, or bleeding at the insertion site. It is suggested that upper limb extremity ergometer exercise can be initiated earlier than mobilization out of bed when the criteria for initiation of exercise and other conditions are met. Only in one case temporary dyspnea occurred after exercise. It is not clear whether the dyspnea that occurred after exercise was induced by the exercise performed because the condition of patients with severe CS changes over time as treatment progresses. Differences in the severity of patient conditions and the use of various devices may have influenced the outcomes, introducing variability to the results. The type of mechanical support or their simultaneous adaptation did not result in such differences. On the other hand, the initial exercise tended to be at a very low intensity, and as the number of exercise sessions increased, the exercise load tended to increase in intensity. The reason for selecting different numbers of sessions per patient was based on individual responses to therapy and clinical progress, which may have influenced the exercise prescription for each case. This is a very important clinical approach and we believe that it is appropriate to start the upper limb extremity ergometer exercise at a low load and then increase the exercise load according to the response during and after the exercise. Upper extremity ergometer exercise can confirm the hemodynamic response to exercise in patients with CS and is useful in evaluating recovery of cardiac function.

In this study, we observed significant changes in the respiratory and heart rates at all time points during the exercise period compared with the pre-exercise period. Although not investigated in this study, upper limb exercises are prone to heart rate and respiratory variabilities (Leicht et al., 2008; Castro et al., 2011). The mechanisms of heart rate and respiratory variability during upper extremity exercise remain to be elucidated; however, the influence of the autonomic nervous system has been suggested (Tulppo et al., 1999). Arm exercises elicit more input from afferent muscle fibers than leg exercises, and the same output causes greater lactate accumulation than leg exercises. Therefore, fatigue is believed to be more noticeable, resulting in greater sympathetic stimulation (Casaburi et al., 1992; Cerny and Ucer, 2004). An increase in the respiratory rate stimulates the sympathetic nervous system, and when sympathetic activity is predominant, the heart rate will increase. In addition, to increase oxygen delivery to muscles throughout the body, both respiratory rate and heart rate increase during exercise. In this way, heart rate and respiration are mutually inducing (Galletly and Larsen, 2001). These autonomic regulatory mechanisms may influence heart rate and respiratory variabilities. Published studies of exercise-induced heart rate and respiratory variability in cardiac patients have mostly investigated the impact of intensities above low to moderate exercise (Brechtel et al., 2007). Considering that this study involved an infinitely low-load exercise and the different pathological conditions of the acute phase of CS, the mechanism may be different from the conventional mechanism.

### 4.1 Study limitations

This study had some limitations. First, critically ill patients who require MCS or MV can experience a high degree of anxiety associated with immobility and the mental stress of being in an extraordinary state. Depressive symptoms, such as agitation and apathy, are also likely to be problematic. Therefore, it is necessary to consider not only the pathological condition but also the psychological state of the patient when considering the indications for exercise. In addition, prolonged ICU stays are prone to delirium complications, which may prevent exercise initiation. Therefore, it is important to create an environment in which mental and cognitive assessments and support are collaborative among multidisciplinary teams so that early rehabilitation can be initiated.

Second, exercise prescription based on objective assessment was not possible because the patients were in the state of the acute phase immediately after the onset of illness; therefore, the exercise load was determined based on vital signs and subjective symptoms. Therefore, it is unclear whether the exercise intensity was adequate for improving cardiopulmonary function. The creation of a standard protocol does remain an issue and should be investigated in the future. Furthermore, the effects of exercise should be studied by randomized controlled trials.

Third, the detailed effects of exercise on the pathophysiology were unclear because we only evaluated vital sign changes, which could be easily monitored in clinical practice. However, we did not investigated other parameters such as a Swan-Ganz catheter pressure or arterial blood gas analysis, which may result in more detailed aspects of hemodynamic or respiratory fluctuations. As this is a feasibility observational study, a cause-and-effect relationship between exercise and adverse events cannot be established. Therefore, the clarification of such a possible causal relationship is an issue for the future.

Furthermore, the study took place in a single center, the inclusion of multiple centers could enhance the external validity of the results.

## 5 Conclusion

Early upper limb extremity ergometer exercises for critically ill patients with CS or at risk for CS resulted in changes in respiratory and heart rates during exercise, but not in systolic or diastolic blood pressure or oxygen saturation. However, no changes were observed after exercise compared to pre-exercise levels, and no exercise-related adverse events occurred. Upper limb extremity ergometer exercise can be initiated earlier in patients with CS or at risk for CS and can be a new tool in the acute phase of physical therapy.

## Data Availability

The raw data supporting the conclusions of this article will be made available by the authors, without undue reservation.

## References

[B1] Baldetti L. Beneduce A. Romagnolo D. Frias A. Gramegna M. Sacchi S. (2023). Impella malrotation within the left ventricle is associated with adverse in-hospital outcomes in cardiogenic shock. JACC Cardiovasc Interv. 16, 739–741. 10.1016/j.jcin.2023.01.020 36990567

[B2] Baran D. A. Grines C. L. Bailey S. Burkhoff D. Hall S. A. Henry T. D. (2019). SCAI clinical expert consensus statement on the classification of cardiogenic shock: this document was endorsed by the American college of cardiology (ACC), the American heart association (AHA), the society of critical care medicine (SCCM), and the society of thoracic surgeons (STS) in April 2019. Catheter Cardiovasc Interv. 94, 29–37. 10.1002/ccd.28329 31104355

[B3] Brechtel L. Lehmann S. Suhl A. Lock J. Wolff R. (2007). Bestimmung von Schwellen der Herzfrequenzvariabilität bei Koronarsportlern - Eine Alternative zur Belastungssteuerung. [Determination of heart rate variability tresholds in heart patients - an alternative to control excercise intensity]. Ger. J. Sports Med. 58 (7/8). 261.

[B4] Casaburi R. Barstow T. J. Robinson T. Wasserman K. (1992). Dynamic and steady-state ventilatory and gas exchange responses to arm exercise. Med. Sci. Sports Exerc 24, 1365–1374. 10.1249/00005768-199212000-00010 1470020

[B5] Castro R. R. Pedrosa S. Nóbrega A. C. (2011). Different ventilatory responses to progressive maximal exercise test performed with either the arms or legs. Clin. (Sao Paulo) 66, 1137–1142. 10.1590/s1807-59322011000700003 PMC3148454 21876964

[B6] Cerny F. J. Ucer C. (2004). Arm work interferes with normal ventilation. Appl. Ergon. 35, 411–415. 10.1016/j.apergo.2004.05.001 15246879

[B7] Cucchi M. Mariani S. De Piero M. E. Ravaux J. M. Kawczynski M. J. Di Mauro M. (2023). Awake extracorporeal life support and physiotherapy in adult patients: a systematic review of the literature. Perfusion 38, 939–958. 10.1177/02676591221096078 35760523 PMC10265312

[B8] da Rosa Pinheiro D. R. Cabeleira M. E. P. da Campo L. A. Gattino L. A. F. de Souza K. S. Dos Santos Burg L. (2021). Upper limbs cycle ergometer increases muscle strength, trunk control and independence of acute stroke subjects: a randomized clinical trial. NeuroRehabilitation 48, 533–542. 10.3233/NRE-210022 33998550

[B9] Eerden S. Dekker R. Hettinga F. J. (2018). Maximal and submaximal aerobic tests for wheelchair-dependent persons with spinal cord injury: a systematic review to summarize and identify useful applications for clinical rehabilitation. Disabil. Rehabil. 40, 497–521. 10.1080/09638288.2017.1287623 28637157

[B10] Ferreira D. D. C. Marcolino M. A. Z. Macagnan F. E. Plentz R. D. M. Kessler A. (2019). Safety and potential benefits of physical therapy in adult patients on extracorporeal membrane oxygenation support: a systematic review. Rev. Bras. Ter. Intensiva 31, 227–239. 10.5935/0103-507X.20190017 31090853 PMC6649220

[B11] Galletly D. C. Larsen P. D. (2001). Cardioventilatory coupling in heart rate variability: methods for qualitative and quantitative determination. Br. J. Anaesth. 87, 827–833. 10.1093/bja/87.6.827 11878682

[B12] Higuchi R. Nanasato M. Furuichi Y. Hosoya Y. Haraguchi G. Takayama M. (2023). Outcomes of octogenarians and nonagenarians in a contemporary Cardiac Care Unit – insights from 2,242 patients admitted between 2019 and 2021. Circ. Rep. 5, 430–436. 10.1253/circrep.CR-23-0078 37969231 PMC10632070

[B13] Ilias N. A. Xian H. Inman C. Martin W. H. 3rd (2009). Arm exercise testing predicts clinical outcome. Am. Heart J. 157, 69–76. 10.1016/j.ahj.2008.09.007 19081399

[B14] Jentzer J. C. van Diepen S. Barsness G. W. Henry T. D. Menon V. Rihal C. S. (2019). Cardiogenic shock classification to predict mortality in the cardiac Intensive Care Unit. J. Am. Coll. Cardiol. 74, 2117–2128. 10.1016/j.jacc.2019.07.077 31548097

[B15] Kawakami D. Fujitani S. Morimoto T. Dote H. Takita M. Takaba A. (2021). Prevalence of post-intensive care syndrome among Japanese intensive care unit patients: a prospective, multicenter, observational J-PICS study. Crit. Care 25, 69. 10.1186/s13054-021-03501-z 33593406 PMC7888178

[B16] Kimura K. Kimura T. Ishihara M. Nakagawa Y. Nakao K. Miyauchi K. (2019). JCS 2018 guideline on diagnosis and treatment of acute coronary syndrome. Circ. J. 83, 1085–1196. 10.1253/circj.CJ-19-0133 30930428

[B17] Kolte D. Khera S. Aronow W. S. Mujib M. Palaniswamy C. Sule S. (2014). Trends in incidence, management, and outcomes of cardiogenic shock complicating ST-elevation myocardial infarction in the United States. J. Am. Heart Assoc. 3, e000590. 10.1161/JAHA.113.000590 24419737 PMC3959706

[B18] Larsen R. T. Christensen J. Tang L. H. Keller C. Doherty P. Zwisler A. D. (2016). A systematic review and meta-analysis comparing cardiopulmonary exercise test values obtained from the arm cycle and the leg cycle respectively in healthy adults. Int. J. Sports Phys. Ther. 11, 1006–1039. 27999717 PMC5159627

[B19] Leicht A. S. Sinclair W. H. Spinks W. L. (2008). Effect of exercise mode on heart rate variability during steady state exercise. Eur. J. Appl. Physiol. 102, 195–204. 10.1007/s00421-007-0574-9 17922138

[B20] Mendelsohn M. E. Overend T. J. Connelly D. M. Petrella R. J. (2008). Improvement in aerobic fitness during rehabilitation after hip fracture. Arch. Phys. Med. Rehabil. 89, 609–617. 10.1016/j.apmr.2007.09.036 18373989

[B21] Needham D. M. Davidson J. Cohen H. Hopkins R. O. Weinert C. Wunsch H. (2012). Improving long-term outcomes after discharge from intensive care unit: report from a stakeholders’ conference. Crit. Care Med. 40, 502–509. 10.1097/CCM.0b013e318232da75 21946660

[B22] Olney C. M. Ferguson J. E. Voss G. Nickel E. Fairhurst S. Bornstein A. S. (2023). Supine arm cycling during the post-flap recovery period for persons with spinal cord injuries: the multi-purpose arm cycle ergometer (M-PACE) safety and pilot testing. J. Spinal Cord. Med. 46, 146–153. 10.1080/10790268.2021.1975082 34726573 PMC9897737

[B23] Paton M. Chan S. Tipping C. J. Stratton A. Serpa Neto A. Lane R. (2023). The effect of mobilization at 6 months after critical illness - meta-analysis. NEJM Evid. 2, EVIDoa2200234. 10.1056/EVIDoa2200234 38320036

[B24] Renner C. Jeitziner M. M. Albert M. Brinkmann S. Diserens K. Dzialowski I. (2023). Guideline on multimodal rehabilitation for patients with post-intensive care syndrome. Crit. Care 27, 301. 10.1186/s13054-023-04569-5 37525219 PMC10392009

[B25] Salna M. Abrams D. Brodie D. (2020). Physical rehabilitation in the awake patient receiving extracorporeal circulatory or gas exchange support. Ann. Transl. Med. 8, 834. 10.21037/atm.2020.03.151 32793679 PMC7396238

[B26] Schaller S. J. Scheffenbichler F. T. Bein T. Blobner M. Grunow J. J. Hamsen U. (2024). Guideline on positioning and early mobilisation in the critically ill by an expert panel. Intensive Care Med. 50, 1211–1227. 10.1007/s00134-024-07532-2 39073582

[B27] TEAM Study Investigators and the ANZICS Clinical Tria, ls Group Hodgson C. L. Bailey M. Bellomo R. Brickell K. Broadley T. (2022). Early active mobilization during mechanical ventilation in the ICU. N. Engl. J. Med. 387, 1747–1758. 10.1056/NEJMoa2209083 36286256

[B28] Tulppo M. P. Mäkikallio T. H. Laukkanen R. T. Huikuri H. V. (1999). Differences in autonomic modulation of heart rate during arm and leg exercise. Clin. Physiol. 19, 294–299. 10.1046/j.1365-2281.1999.00180.x 10451789

[B29] Unoki T. Hayashida K. Kawai Y. Taito S. Ando M. Iida Y. (2023). Japanese clinical practice guidelines for rehabilitation in critically ill patients 2023 (J-ReCIP 2023). J. Intensive Care 11, 47. 10.1186/s40560-023-00697-w 37932849 PMC10629099

[B30] Zwierska I. Walker R. D. Choksy S. A. Male J. S. Pockley A. G. Saxton J. M. (2005). Upper-vs lower-limb aerobic exercise rehabilitation in patients with symptomatic peripheral arterial disease: a randomized controlled trial. J. Vasc. Surg. 42, 1122–1130. 10.1016/j.jvs.2005.08.021 16376202

